# Supplementary material for: Rational design of a JAK1-selective siRNA inhibitor for the modulation of autoimmunity in the skin
Source: Nat Commun. 2023 Nov 4;14:7099. doi: 10.1038/s41467-023-42714-4 (PMC10625637; doi:10.1038/s41467-023-42714-4)
Supplement: Supplementary file 1 — Supplementary information [file 41467_2023_42714_MOESM1_ESM.pdf]

Supplementary Information

Rational design of a JAK1-selective siRNA inhibitor for the modulation of autoimmunity in the skin

Qi Tang[1,2], Hassan H. Fakih[2], Mohammad Zain Ul Abideen[2], Samuel R. Hildebrand[2], Khashayar Afshari[1], Katherine Y. Gross[2], Jacquelyn Sousa[2], Allison S. Maebius[3], Christina Bartholdy[4], Pia Pernille Søgaard[4], Malene Jackerott[4], Vignesh Hariharan[2], Ashley Summers[2], Xueli Fan[1], Ken Okamura[1], Kathryn R. Monopoli[2,5], David A. Cooper[2,3], Dimas Echeverria[2], Brianna Bramato[2], Nicholas McHugh[2], Raymond C. Furgal[2], Karen Dresser[6], Sarah J. Winter[2], Annabelle Biscans[2], Jane Chuprin[1], Nazgol-Sadat Haddadi[1], Shany Sherman[1], Ümmügülsüm Yıldız-Altay[1], Mehdi Rashighi[1], Jillian M. Richmond[1], Claire Bouix-Peter[7], Carine Blanchard[7], Adam Clauss[4], Julia F. Alterman[2] *, Anastasia Khvorova[2,3] *, and John E. Harris[1] *

[1] Department of Dermatology, University of Massachusetts Chan Medical School, Worcester, MA 01605, USA.
[2] RNA Therapeutics Institute, University of Massachusetts Chan Medical School, Worcester, MA 01605, USA.
[3] Program in Molecular Medicine, University of Massachusetts Chan Medical School, Worcester, MA 01605, USA.
[4] LEO Pharma A/S, Industriparken 55, 2750 Ballerup, Denmark.
[5] Bioinformatics and Computational Biology Program, Worcester Polytechnic Institute, Worcester, MA 01609, USA.
[6] Department of Pathology, University of Massachusetts Chan Medical School, Worcester, MA 01605, USA.
[7] Aldena Therapeutics, London E1 6RA, UK.

***** Correspondence should be addressed to:
John.Harris@umassmed.edu, Anastasia.Khvorova@umassmed.edu, or Julia.Alterman@umassmed.edu

Content:

Supplementary Table 1. siRNA sequences and the modification patterns used for *in vitro* screening.
Supplementary Figure 1. 3033 targeting site and surrounding regions in JAK1 mRNA transcripts are fully conserved across human, non-human primates, and mouse.
Supplementary Figure 2. Quantification and visualization of si3033 at skin local to the injection site and other major organs.
Supplementary Figure 3. Local left footpad injection of si3033 does not cross-deliver si3033 to the right footpad skin.
Supplementary Figure 4. Flow cytometry gating of autoreactive CD8$^+$ PMEL T cells in footpad skin of vitiligo mice.
Supplementary Figure 5. si3033 prevents skin pigmentation in an autoreactive CD8$^+$ T cell-mediated vitiligo mouse model.
Supplementary Figure 6. si3033 is cross-reactive to rat Jak1 mRNA transcripts *in vivo*.
Supplementary Figure 7. Gating strategy for human skin cell types.
Supplementary Figure 8. Boxplots of 3 clusters of genes for which the interaction term between IFN-γ stimulation and JAK1 knockdown was statistically significant in human skin explant.
Supplementary Figure 9. Boxplots of expression for genes involved in IFN-γ stimulation.

Supplementary Table 1 | siRNA sequences and the modification patterns used for *in vitro* screening.

§ *JAK1*_NM_001321852_883	ø P(mU)#(fC)#(mU)(mU)(mA)(fU)(mU)(mC)(mA)(mA)(mU)(mG)(mU)#(fU)#(mU)#(fC)#(mU)#(mG)#(mG)#(fA)
	¶ (mA)#(mA)#(mA)(mC)(fA)(fU)(fU)(mG)(fA)(mA)(mU)(mA)(mA)#(mG)#(mA)-TegChol
*JAK1*_NM_001321852_1048	P(mU)#(fA)#(mC)(mC)(mG)(fU)(mA)(mA)(mU)(mG)(mU)(mU)(mU)#(fU)#(mG)#(fU)#(mC)#(mA)#(mA)#(fA)
	(mC)#(mA)#(mA)(mA)(fA)(fC)(fA)(mU)(fU)(mA)(mC)(mG)(mG)#(mU)#(mA)-TegChol
*JAK1*_NM_001321852_1194	P(mU)#(fA)#(mC)(mA)(mU)(fU)(mU)(mG)(mG)(mU)(mU)(mU)(mA)#(fU)#(mG)#(fC)#(mC)#(mU)#(mC)#(fC)
	(mC)#(mA)#(mU)(mA)(fA)(fA)(fC)(mC)(fA)(mA)(mA)(mU)(mG)#(mU)#(mA)-TegChol
*JAK1*_NM_001321852_2747	P(mU)#(fC)#(mA)(mA)(mG)(fC)(mU)(mU)(mA)(mU)(mU)(mA)(mA)#(fU)#(mG)#(fU)#(mC)#(mU)#(mC)#(fU)
	(mC)#(mA)#(mU)(mU)(fA)(fA)(fU)(mA)(fA)(mG)(mC)(mU)(mU)#(mG)#(mA)-TegChol
*JAK1*_NM_001321852_3379	P(mU)#(fA)#(mU)(mA)(mA)(fA)(mA)(mU)(mU)(mU)(mA)(mG)(mA)#(fU)#(mU)#(fG)#(mC)#(mA)#(mU)#(fU)
	(mA)#(mA)#(mU)(mC)(fU)(fA)(fA)(mA)(fU)(mU)(mU)(mU)(mA)#(mU)#(mA)-TegChol
*JAK1*_NM_001321852_3908	P(mU)#(fA)#(mA)(mA)(mU)(fA)(mU)(mU)(mU)(mU)(mG)(mG)(mU)#(fU)#(mG)#(fU)#(mC)#(mA)#(mU)#(fU)
	(mC)#(mA)#(mA)(mC)(fC)(fA)(fA)(mA)(fA)(mU)(mA)(mU)(mU)#(mU)#(mA)-TegChol
*JAK1*_NM_001321852_4019	P(mU)#(fU)#(mC)(mA)(mA)(fC)(mU)(mC)(mC)(mU)(mC)(mG)(mU)#(fU)#(mU)#(fU)#(mC)#(mA)#(mA)#(fA)
	(mA)#(mA)#(mA)(mC)(fG)(fA)(fG)(mG)(fA)(mG)(mU)(mU)(mG)#(mA)#(mA)-TegChol
*JAK1*_NM_001321852_4034	P(mU)#(fC)#(mA)(mG)(mA)(fU)(mA)(mU)(mU)(mA)(mU)(mU)(mU)#(fU)#(mG)#(fG)#(mU)#(mC)#(mA)#(fA)
	(mC)#(mA)#(mA)(mA)(fA)(fU)(fA)(mA)(fU)(mA)(mU)(mC)(mU)#(mG)#(mA)-TegChol
*JAK1*_NM_001321852_4348	P(mU)#(fG)#(mC)(mA)(mA)(fA)(mU)(mU)(mA)(mU)(mC)(mU)(mA)#(fU)#(mU)#(fC)#(mC)#(mA)#(mC)#(fA)
	(mA)#(mA)#(mU)(mA)(fG)(fA)(fU)(mA)(fA)(mU)(mU)(mU)(mG)#(mC)#(mA)-TegChol
*JAK1*_NM_001321852_4470	P(mU)#(fA)#(mU)(mA)(mC)(fA)(mU)(mU)(mG)(mA)(mC)(mG)(mU)#(fU)#(mU)#(fC)#(mU)#(mU)#(mA)#(fA)
	(mA)#(mA)#(mA)(mC)(fG)(fU)(fC)(mA)(fA)(mU)(mG)(mU)(mA)#(mU)#(mA)-TegChol
*JAK1*_NM_001321852_4889	P(mU)#(fC)#(mA)(mA)(mC)(fU)(mU)(mC)(mA)(mU)(mU)(mG)(mC)#(fU)#(mG)#(fC)#(mC)#(mA)#(mC)#(fU)
	(mC)#(mA)#(mG)(mC)(fA)(fA)(fU)(mG)(fA)(mA)(mG)(mU)(mU)#(mG)#(mA)-TegChol
*JAK1*_NM_001321852_4904	P(mU)#(fA)#(mA)(mC)(mA)(fA)(mA)(mU)(mU)(mU)(mA)(mA)(mA)#(fU)#(mG)#(fG)#(mC)#(mA)#(mA)#(fC)
	(mC)#(mA)#(mU)(mU)(fU)(fA)(fA)(mA)(fU)(mU)(mU)(mG)(mU)#(mU)#(mA)-TegChol
*JAK*_NM_001321852_212	P(mU)#(fG)#(mA)(mU)(mA)(fC)(mU)(mG)(mC)(mA)(mU)(mU)(mU)#(fA)#(mU)#(fU)#(mC)#(mA)#(mG)#(fC)
	(mA)#(mU)#(mA)(mA)(fA)(fU)(fG)(mC)(fA)(mG)(mU)(mA)(mU)#(mC)#(mA)-TegChol
*JAK1*_NM_001321852_214	P(mU)#(fU)#(mA)(mG)(mA)(fU)(mA)(mC)(mU)(mG)(mC)(mA)(mU)#(fU)#(mU)#(fA)#(mU)#(mU)#(mC)#(fA)
	(mA)#(mA)#(mA)(mU)(fG)(fC)(fA)(mG)(fU)(mA)(mU)(mC)(mU)#(mA)#(mA)-TegChol
*JAK1*_NM_001321852_964	P(mU)#(fC)#(mU)(mU)(mG)(fU)(mU)(mG)(mU)(mU)(mA)(mA)(mA)#(fU)#(mU)#(fC)#(mC)#(mU)#(mU)#(fU)
	(mA)#(mA)#(mU)(mU)(fU)(fA)(fA)(mC)(fA)(mA)(mC)(mA)(mA)#(mG)#(mA)-TegChol
*JAK1*_NM_001321852_1067	P(mU)#(fA)#(mA)(mG)(mU)(fC)(mU)(mC)(mA)(mA)(mA)(mU)(mA)#(fU)#(mU)#(fU)#(mC)#(mA)#(mG)#(fC)
	(mA)#(mA)#(mU)(mA)(fU)(fU)(fU)(mG)(fA)(mG)(mA)(mC)(mU)#(mU)#(mA)-TegChol
*JAK1*_NM_001321852_1226	P(mU)#(fU)#(mC)(mA)(mG)(fU)(mU)(mU)(mA)(mU)(mU)(mU)(mU)#(fU)#(mU)#(fU)#(mC)#(mC)#(mU)#(fU)
	(mA)#(mA)#(mA)(mA)(fA)(fA)(fU)(mA)(fA)(mA)(mC)(mU)(mG)#(mA)#(mA)-TegChol
*JAK1*_NM_001321852_1240	P(mU)#(fC)#(mA)(mG)(mU)(fU)(mU)(mU)(mU)(mU)(mC)(mC)(mG)#(fC)#(mU)#(fU)#(mC)#(mA)#(mG)#(fU)
	(mA)#(mG)#(mC)(mG)(fG)(fA)(fA)(mA)(fA)(mA)(mA)(mC)(mU)#(mG)#(mA)-TegChol
*JAK1*_NM_001321852_1242	P(mU)#(fU)#(mC)(mC)(mA)(fG)(mU)(mU)(mU)(mU)(mU)(mU)(mC)#(fC)#(mG)#(fC)#(mU)#(mU)#(mC)#(fA)
	(mC)#(mG)#(mG)(mA)(fA)(fA)(fA)(mA)(fA)(mC)(mU)(mG)(mG)#(mA)#(mA)-TegChol
*JAK1*_NM_001321852_1345	P(mU)#(fC)#(mA)(mC)(mA)(fG)(mA)(mC)(mU)(mC)(mC)(mU)(mU)#(fU)#(mA)#(fU)#(mU)#(mA)#(mC)#(fA)
	(mU)#(mA)#(mA)(mA)(fG)(fG)(fA)(mG)(fU)(mC)(mU)(mG)(mU)#(mG)#(mA)-TegChol
*JAK1*_NM_001321852_2063	P(mU)#(fG)#(mG)(mA)(mU)(fC)(mA)(mC)(mU)(mU)(mU)(mU)(mA)#(fU)#(mC)#(fU)#(mU)#(mC)#(mU)#(fU)
	(mG)#(mA)#(mU)(mA)(fA)(fA)(fA)(mG)(fU)(mG)(mA)(mU)(mC)#(mC)#(mA)-TegChol
*JAK1*_NM_001321852_3033	P(mU)#(fC)#(mA)(mG)(mA)(fU)(mU)(mC)(mC)(mU)(mU)(mU)(mG)#(fU)#(mA)#(fC)#(mU)#(mU)#(mC)#(fA)
	(mU)#(mA)#(mC)(mA)(fA)(fA)(fG)(mG)(fA)(mA)(mU)(mC)(mU)#(mG)#(mA)-TegChol
*JAK1*_NM_001321852_3232	P(mU)#(fA)#(mA)(mG)(mG)(fA)(mC)(mA)(mU)(mU)(mU)(mC)(mU)#(fU)#(mG)#(fC)#(mU)#(mG)#(mC)#(fC)
	(mC)#(mA)#(mA)(mG)(fA)(fA)(fA)(mU)(fG)(mU)(mC)(mC)(mU)#(mU)#(mA)-TegChol
*JAK1*_NM_001321852_3668	P(mU)#(fC)#(mU)(mU)(mA)(fU)(mU)(mU)(mU)(mA)(mA)(mA)(mA)#(fG)#(mU)#(fG)#(mC)#(mU)#(mU)#(fC)
	(mA)#(mC)#(mU)(mU)(fU)(fU)(fA)(mA)(fA)(mA)(mU)(mA)(mA)#(mG)#(mA)-TegChol
*Jak1*_NM_146145_302	P(mU)#(fG)#(mC)(mA)(mU)(fU)(mU)(mA)(mU)(mU)(mC)(mG)(mG)#(fU)#(mU)#(fG)#(mU)#(mC)#(mC)#(fA)
	(mA)#(mA)#(mC)(mC)(fG)(fA)(fA)(mU)(fA)(mA)(mA)(mU)(mG)#(mC)#(mA)-TegChol
*Jak1*_NM_146145_1027	P(mU)#(fA)#(mA)(mC)(mA)(fU)(mU)(mA)(mU)(mU)(mU)(mA)(mU)#(fU)#(mC)#(fG)#(mC)#(mA)#(mU)#(fC)
	(mG)#(mA)#(mA)(mU)(fA)(fA)(fA)(mU)(fA)(mA)(mU)(mG)(mU)#(mU)#(mA)-TegChol
*Jak1*_NM_146145_1144	P(mU)#(fU)#(mC)(mC)(mA)(fU)(mA)(mA)(mU)(mG)(mU)(mU)(mU)#(fU)#(mG)#(fU)#(mC)#(mA)#(mA)#(fA)
	(mC)#(mA)#(mA)(mA)(fA)(fC)(fA)(mU)(fU)(mA)(mU)(mG)(mG)#(mA)#(mA)-TegChol
*Jak1*_NM_146145_1291	P(mU)#(fA)#(mA)(mC)(mA)(fA)(mC)(mA)(mU)(mU)(mU)(mG)(mG)#(fU)#(mU)#(fU)#(mC)#(mU)#(mG)#(fC)
	(mA)#(mA)#(mC)(mC)(fA)(fA)(fA)(mU)(fG)(mU)(mU)(mG)(mU)#(mU)#(mA)-TegChol
*Jak1*_NM_146145_3214	P(mU)#(fG)#(mU)(mU)(mC)(fU)(mU)(mA)(mU)(mU)(mC)(mU)(mU)#(fU)#(mG)#(fG)#(mC)#(mA)#(mG)#(fA)
	(mC)#(mA)#(mA)(mA)(fG)(fA)(fA)(mU)(fA)(mA)(mG)(mA)(mA)#(mC)#(mA)-TegChol
*Jak1*_NM_146145_3460	P(mU)#(fC)#(mU)(mG)(mG)(fA)(mU)(mU)(mA)(mA)(mA)(mC)(mA)#(fU)#(mU)#(fC)#(mC)#(mG)#(mG)#(fA)
	(mA)#(mA)#(mU)(mG)(fU)(fU)(fU)(mA)(fA)(mU)(mC)(mC)(mA)#(mG)#(mA)-TegChol
*Jak1*_NM_146145_3785	P(mU)#(fG)#(mG)(mA)(mA)(fU)(mU)(mU)(mA)(mA)(mA)(mU)(mG)#(fU)#(mU)#(fG)#(mU)#(mU)#(mC)#(fA)
	(mA)#(mA)#(mC)(mA)(fU)(fU)(fU)(mA)(fA)(mA)(mU)(mU)(mC)#(mC)#(mA)-TegChol
*Jak1*_NM_146145_3990	P(mU)#(fC)#(mC)(mA)(mA)(fA)(mU)(mU)(mU)(mA)(mA)(mG)(mA)#(fU)#(mG)#(fU)#(mU)#(mA)#(mC)#(fA)
	(mC)#(mA)#(mU)(mC)(fU)(fU)(fA)(mA)(fA)(mU)(mU)(mU)(mG)#(mG)#(mA)-TegChol
*Jak1*_NM_146145_4620	P(mU)#(fA)#(mA)(mA)(mG)(fC)(mU)(mG)(mU)(mU)(mG)(mA)(mU)#(fU)#(mA)#(fC)#(mC)#(mC)#(mA)#(fG)
	(mU)#(mA)#(mA)(mU)(fC)(fA)(fA)(mC)(fA)(mG)(mC)(mU)(mU)#(mU)#(mA)-TegChol
*Jak1*_NM_146145_4699	P(mU)#(fA)#(mC)(mA)(mA)(fG)(mU)(mA)(mU)(mU)(mA)(mA)(mA)#(fC)#(mA)#(fG)#(mC)#(mA)#(mU)#(fU)
	(mU)#(mG)#(mU)(mU)(fU)(fA)(fA)(mU)(fA)(mC)(mU)(mU)(mG)#(mU)#(mA)-TegChol
*Jak1*_NM_146145_4729	P(mU)#(fA)#(mA)(mA)(mA)(fG)(mG)(mG)(mC)(mU)(mC)(mA)(mA)#(fA)#(mA)#(fG)#(mG)#(mU)#(mG)#(fA)
	(mU)#(mU)#(mU)(mU)(fG)(fA)(fG)(mC)(fC)(mC)(mU)(mU)(mU)#(mU)#(mA)-TegChol
*Jak1*_NM_146145_4771	P(mU)#(fC)#(mA)(mA)(mC)(fU)(mG)(mU)(mA)(mC)(mU)(mG)(mU)#(fU)#(mG)#(fC)#(mU)#(mA)#(mC)#(fU)
	(mC)#(mA)#(mA)(mC)(fA)(fG)(fU)(mA)(fC)(mA)(mG)(mU)(mU)#(mG)#(mA)-TegChol

§ Target name_Accession number_mRNA targeting site; ø siRNA antisense strand sequence and modifications: m = 2'-*O*-methyl; f = 2'-Fluoro; # = Phosphorothioate; P = 5'-Phosphate; ¶ siRNA sense strand sequence and modifications: TegChol = Teg linker + 3'-Cholesterol.

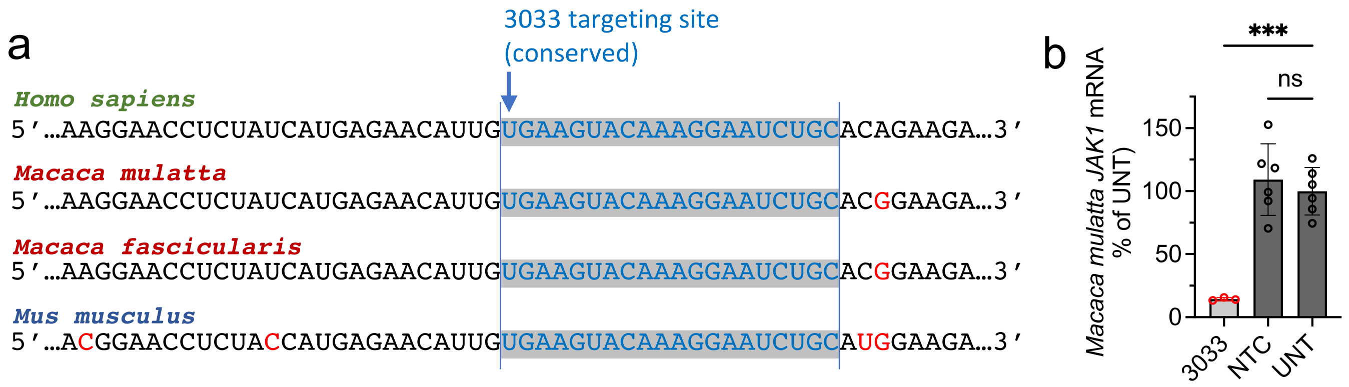

Supplementary Figure 1 | 3033 targeting site and surrounding regions in JAK1 mRNA transcripts are fully conserved across human, non-human primates, and mouse. a. The conserved regions of mRNA transcripts in common experimental species allow the design of siRNAs that are cross-reactive, which simplifies the process of preclinical development. NCBI accession numbers of JAK1 mRNA: *Homo sapiens* (NM_001321852), *Macaca mulatta* (NM: 001257909), *Macaca fascicularis* (XM_005543157), and *Mus musculus* (NM_146145). Red colored bases represent non-conserved mRNA bases across the listed species that may alter the cross-activity of siRNA. **b.** *Macaca mulatta* DBS-FRhL-2 cells were treated with fully modified cholesterol-conjugated 3033 at 1.5 μM for 72 h. JAK1 mRNA level was measured by the QuantiGene 2.0 assay. UNT, untreated control; NTC, non-targeting control siRNA. (*n* = 3 or 6, mean ± s.d.; one-way ANOVA, ****P* < 0.001; ns: not significant).

a

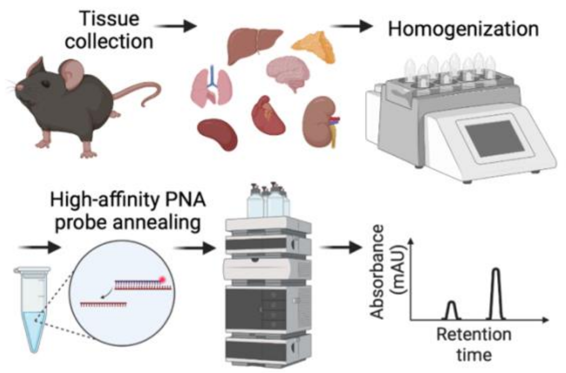

c

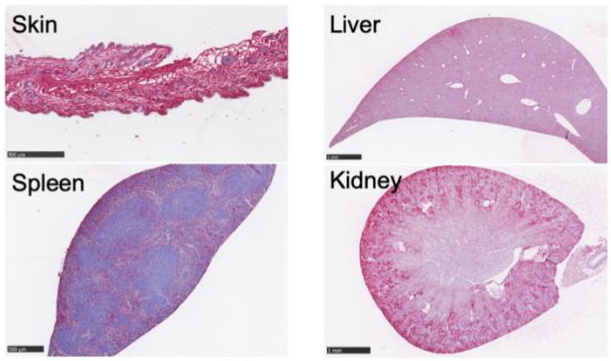

b

Tissue	ng/mg tissue	Standard deviation
Skin (injection site)	262.1	112.3
Liver	230.6	83.4
Spleen	96.1	17.5
Kidney	89.8	16.7
Adrenal gland	67.0	11.7
Duodenum	36.6	10.3
Heart	36.0	3.0
Lung	32.2	8.9
Uterus	21.7	13.3
Bladder	12.4	2.7
Stomach	11.5	2.4
Thymus	6.7	2.9
Pancreas	5.7	2.5
Fat	5.0	1.1
Muscle	4.1	1.5
Skin (distal)	3.6	1.1
Brain	-2.0	3.3

d

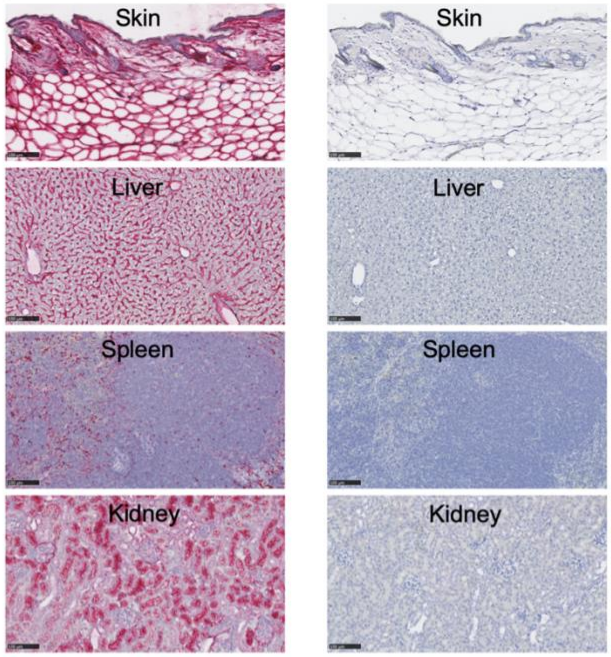

e

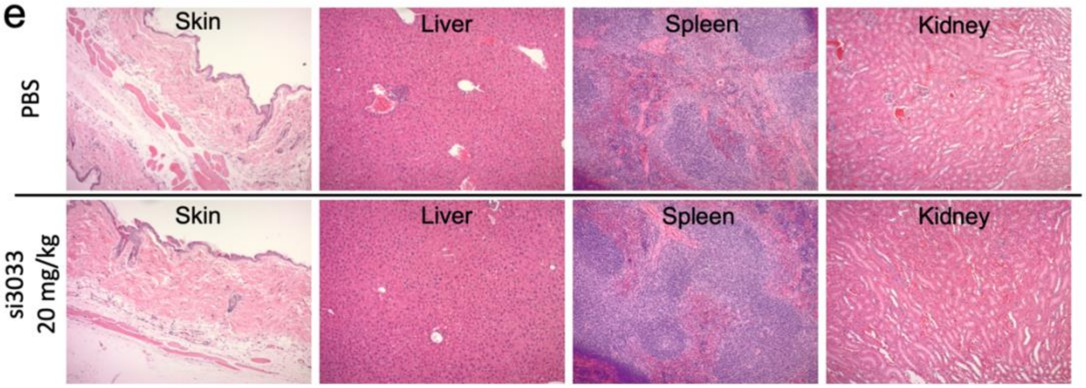

Supplementary Figure 2 | **Quantification and visualization of si3033 at skin local to the injection site and other major organs.** The accumulation of si3033 (20 mg/kg, single dose) at 1 week post subcutaneous injection was quantified by peptide nucleic acid (PNA) assay and visualized by miRNAscope assay. **a.** Schematic of PNA-based quantification of siRNA in tissue samples. A high affinity Cy3-fluorescent oligonucleotide probe was annealed to the complementary antisense strand of si3033 and analyzed by reverse-phase HPLC equipped with fluorescence detector. **b.** Quantification of antisense strands of si3033 in a total of 17 tested tissues. **c.** Representative miRNAscope images of si3033 staining in skin local to the injection site (bar: 500 µm), liver (bar: 1 mm), spleen (bar: 500 µm), kidney (bar: 1 mm), si3033 was stained by red chromogen and images were taken on Nanozoomer 2.0 HT scanner. **d.** 20x magnification of si3033 in local skin, liver, spleen, and kidney; Negative control: si3033 signal was undetectable in siNTC injected mouse samples; bar: 100 µm. **e.** H&E staining of top 4 tissues with high accumulation of si3033; 7 days post S.C. injection at 20 mg/kg dose (n=3); 100x magnification.

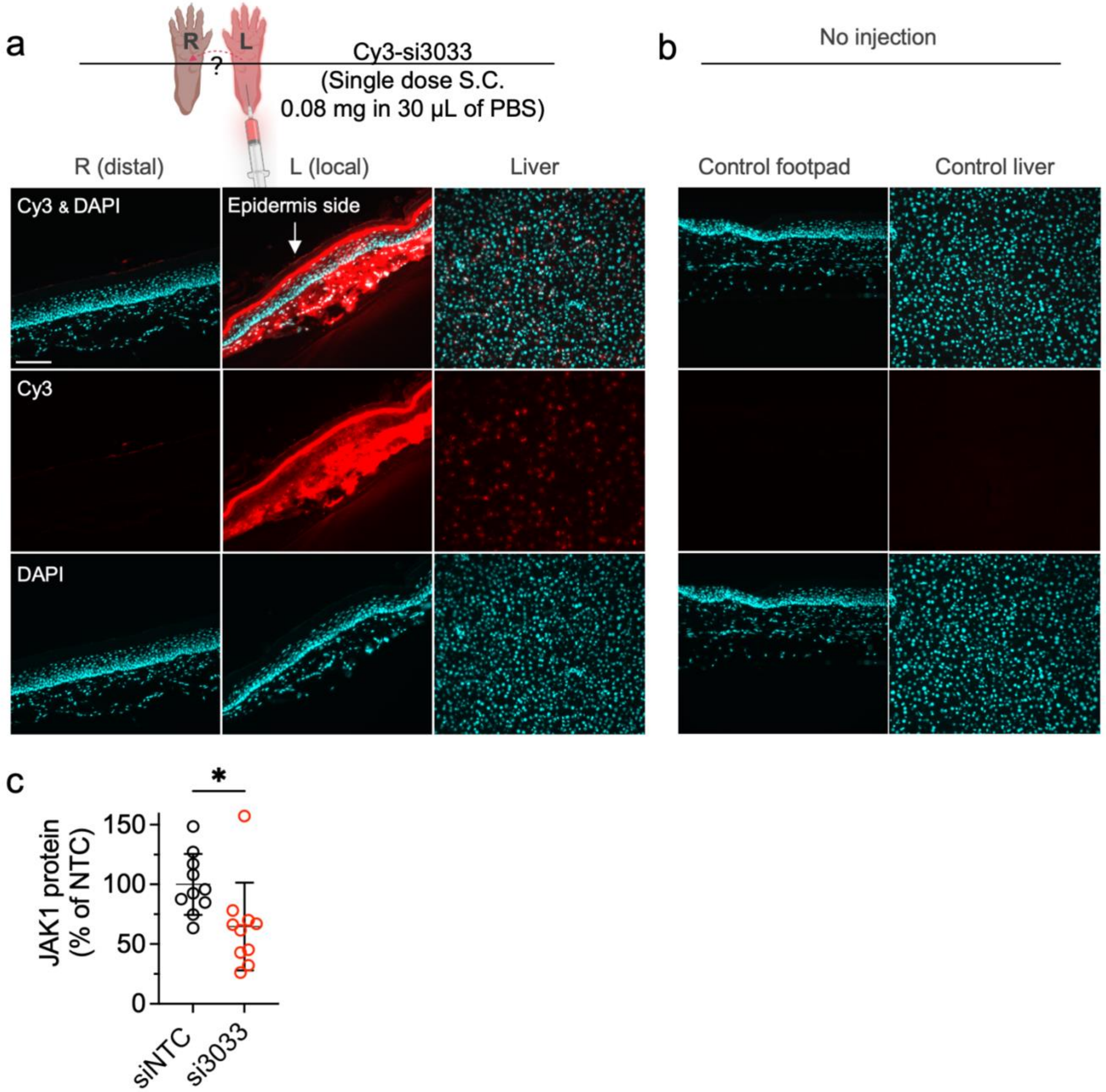

Supplementary Figure 3 | **Local left footpad injection of si3033 does not cross-deliver si3033 to the right footpad skin. a.** Fluorescently (Cy3) labeled si3033 was subcutaneously injected into footpad skin (*n* = 3). Footpad skin and liver were collected 48 h post injection and fresh-frozen in optimal cutting temperature (O.C.T.) compound for tissue sectioning; nuclei were stained with DAPI and si3033 was visualized by fluorescent microscope; bar: 100 μm. si3033 accumulates in liver as expected, the therapeutic dose used in vitiligo model mice did not cross-accumulate in right footpad skin. **b.** Background control of fluorescent images from mice without injections (*n* = 1). **c.** Jak1 protein silencing in the footpad skin (Right vs. left pad) at 2 weeks post 4 mg/kg siRNA injection. Mouse Jak1 protein expression was measured using western capillary ProteinSimple assay (*n* = 10, mean ± s.d.; paired t test, *$P < 0.05$).

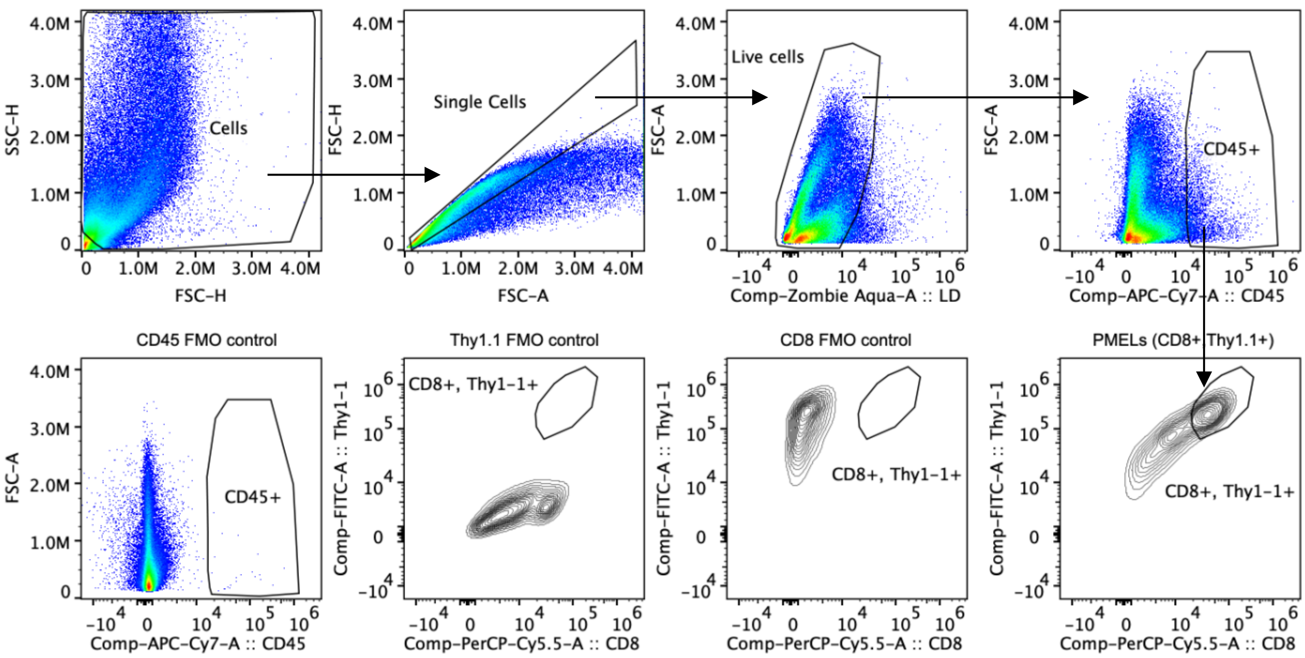

Supplementary Figure 4 | Flow cytometry gating of autoreactive CD8^{+} PMEL T cells in footpad skin of vitiligo mice. A single cell suspension of footpad skin was stained with CD45, CD8, and Thy1.1 markers to identify donor specific PMEL CD8^{+} T cells. Gating strategy: single cells were gated by FSC-H/SSC-H followed by FSC-A/FSC-H gating. Dead cells were excluded by Zombie Aqua staining. Stringent gating of hematopoietic cells (CD45^{+}) was applied to identify the population containing CD8^{+} and Thy1.1^{+} PMEL CD8^{+} T cells originated from donor mice. Fluorescence minus one (FMO) controls were used to define the CD45^{+}, CD8^{+}, and Thy1.1^{+} subset.

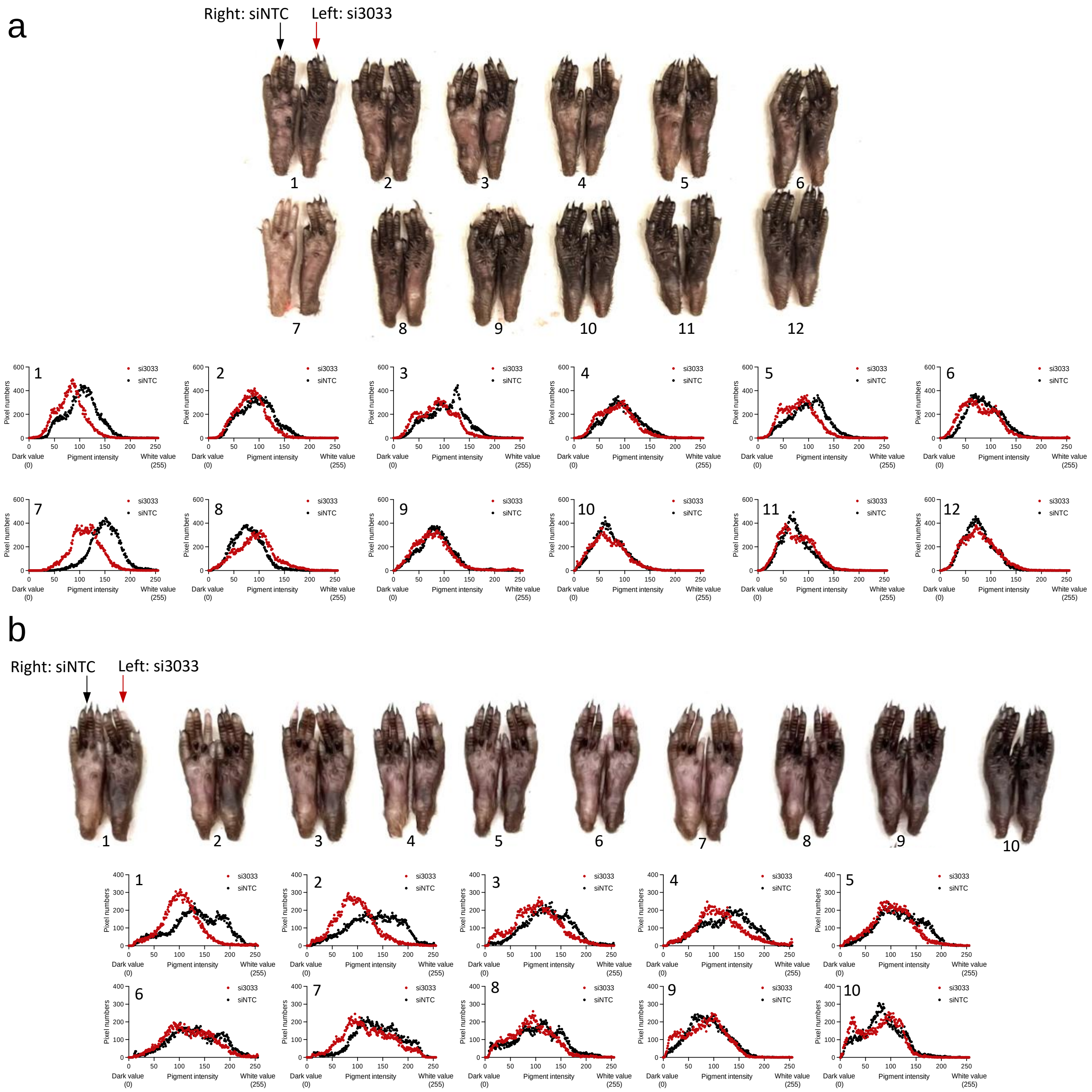

Supplementary Figure 5 | si3033 prevents skin pigmentation in an autoreactive CD8⁺ T cell-mediated vitiligo mouse model. a. Quantification of pigment intensity of the treated footpads (*n* =12; right: siNTC, left si3033). Pigment distribution profiles were analyzed using histogram analysis in Image J Fiji software and presented as the pixel numbers of each intensity value in the range of 0-255 (0: dark value, and 255: white value). **b.** The phenotype of si3033 treatment was reproduced in an independent experiment (*n* =10). As the disease progression (depigmentation) has inter-individual variability, the pigment distribution profile of si3033 treated footpad was visualized by aligning to the profile the siNTC treated footpad in each mouse.

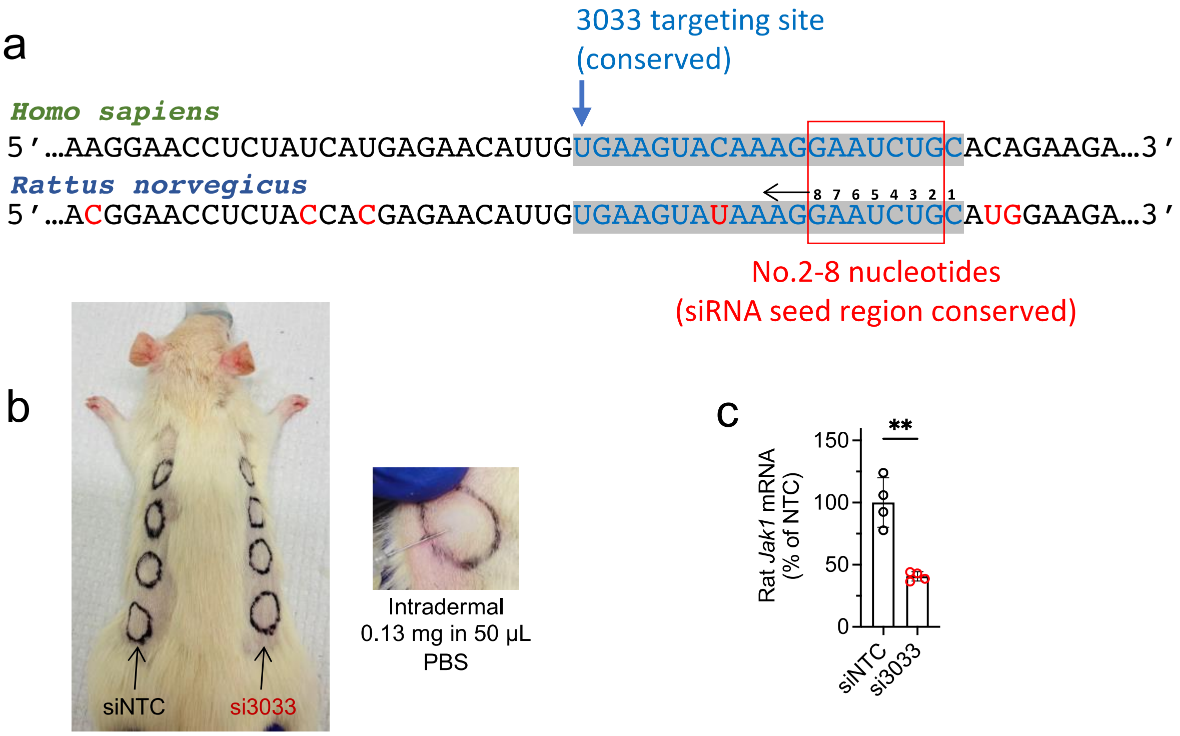

Supplementary Figure 6 | si3033 is cross-reactive to rat Jak1 mRNA transcripts *in vivo*. a. si3033 targeting site is highly conserved between human and rat JAK1 mRNA transcripts. The seed region of si3033 antisense strand has full complementarity to its mRNA targeting sites of the two species. NCBI accession numbers of JAK1 mRNA: *Homo sapiens* (NM_001321852) and *Rattus norveqicus* (NM_053466). Red colored bases represent non-conserved mRNA bases across the listed species that may alter the cross-activity of siRNA. **b.** Female Lewis *Rat* dorsal skin was shaved under anesthesia (isoflurane gas vaporizer system) and intradermally treated with 0.13 mg (in 50 µL PBS) of si3033 (right dorsal side) and siNTC (left dorsal side). The siRNA injection sites were marked with circles (in approximately 1 cm diameter) to precisely locate the treated sites for skin sampling. 1 week after injection, 8-mm diameter biopsy punches were collected into RNAlater® for overnight and were homogenized in 600 µL of QuantiGene™ Homogenizing Solution. Rat Jak1 mRNA level was measured by the QuantiGene 2.0 assay and the expression of Jak1 mRNA was normalized to the expression of Actb as a housekeeping gene (n = 4, mean ± s.d.; unpaired t test, $^{**}P < 0.01$).

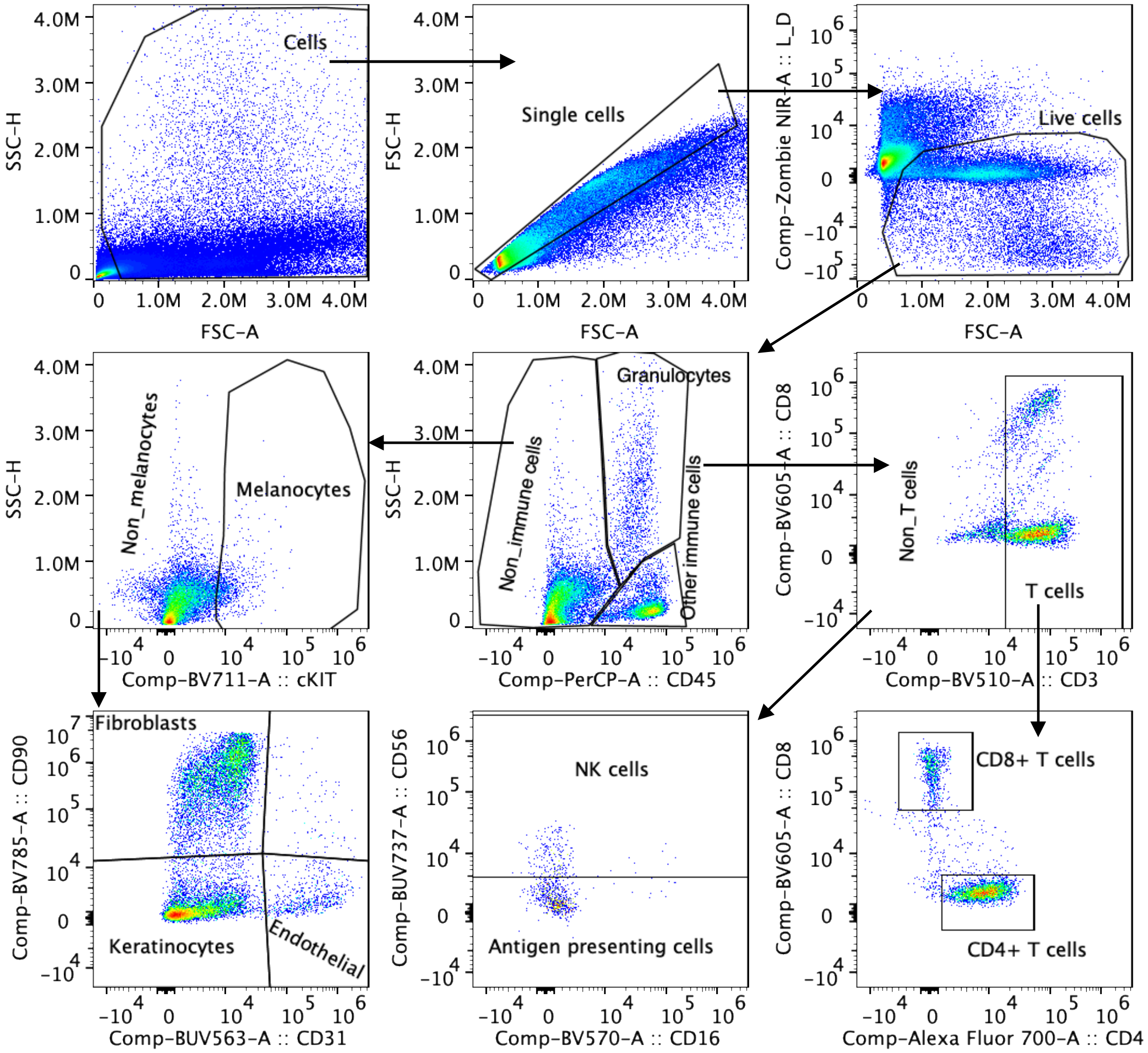

Supplementary Figure 7 | Gating strategy for human skin cell types. The accumulation of Cy3-labeled si3033 in major cell types of human skin was analyzed using Cytex Aurora cytometer and the data processed by FlowJo 10 software. Cells were gated by FSC-A/SSC-H followed by FSC-A/FSC-H for single cells. Dead cells were excluded with Zombie NIR staining. Granulocytes were identified using CD45 gating against SSC-H. The CD45$^-$ population was defined as non-immune cells and further gated into melanocytes and non-melanocytes using c-kit (CD117) as a marker. The non-melanocyte subset was stained with anti-CD31 and anti-CD90 antibodies; defining CD31$^+$ CD90$^-$ subset as endothelial cells, CD31$^+$ CD90$^-$ subset as fibroblasts, and the CD31$^-$ CD90$^-$ population as keratinocytes. The CD45$^+$ population was further gated using anti-CD3 antibody to identify T lymphocytes, the CD3+ population was further stained with anti-CD4 and anti-CD8 antibodies and gated to distinguish the CD4$^+$ and CD8$^+$ subsets, the CD3$^-$ non-T cells population was gated using anti-CD16 and anti-CD56 antibodies to identify NK cells and antigen presenting cells (APCs).

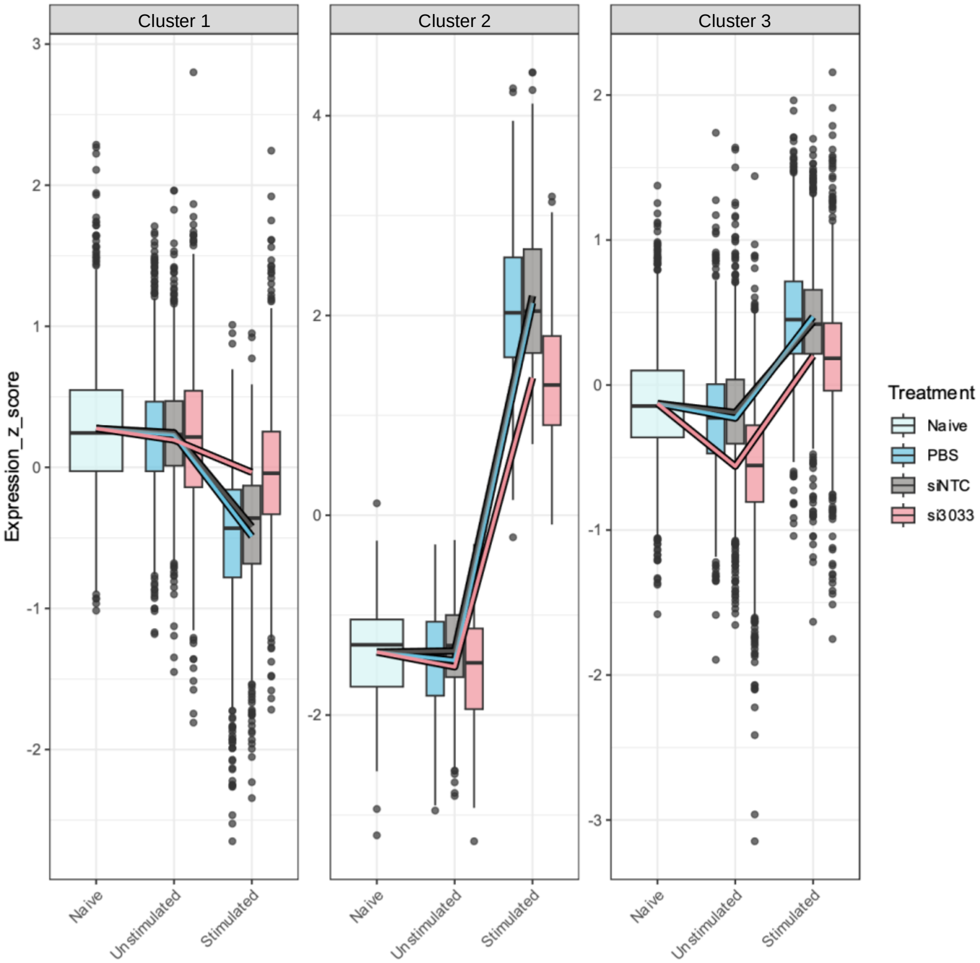

Supplementary Figure 8 | Boxplots of 3 clusters of genes for which the interaction term between IFN-γ stimulation and JAK1 knockdown was statistically significant in human skin explant. Human skin biopsies were injected with PBS, 0.13 mg siNTC, or si3033 and cultured for 4 days *ex vivo* (unstimulated; naive: non-injected control); IFN-γ signaling in a separate set of samples was induced with 10 ng/mL of recombinant IFN-γ and 10 ng/mL of TNF-α for 24 h (stimulated). Expression levels of all genes within each cluster were plotted as boxplots with the mean of all genes in each condition represented by a colored line to allow easy visualization of the overall expression profile trend.

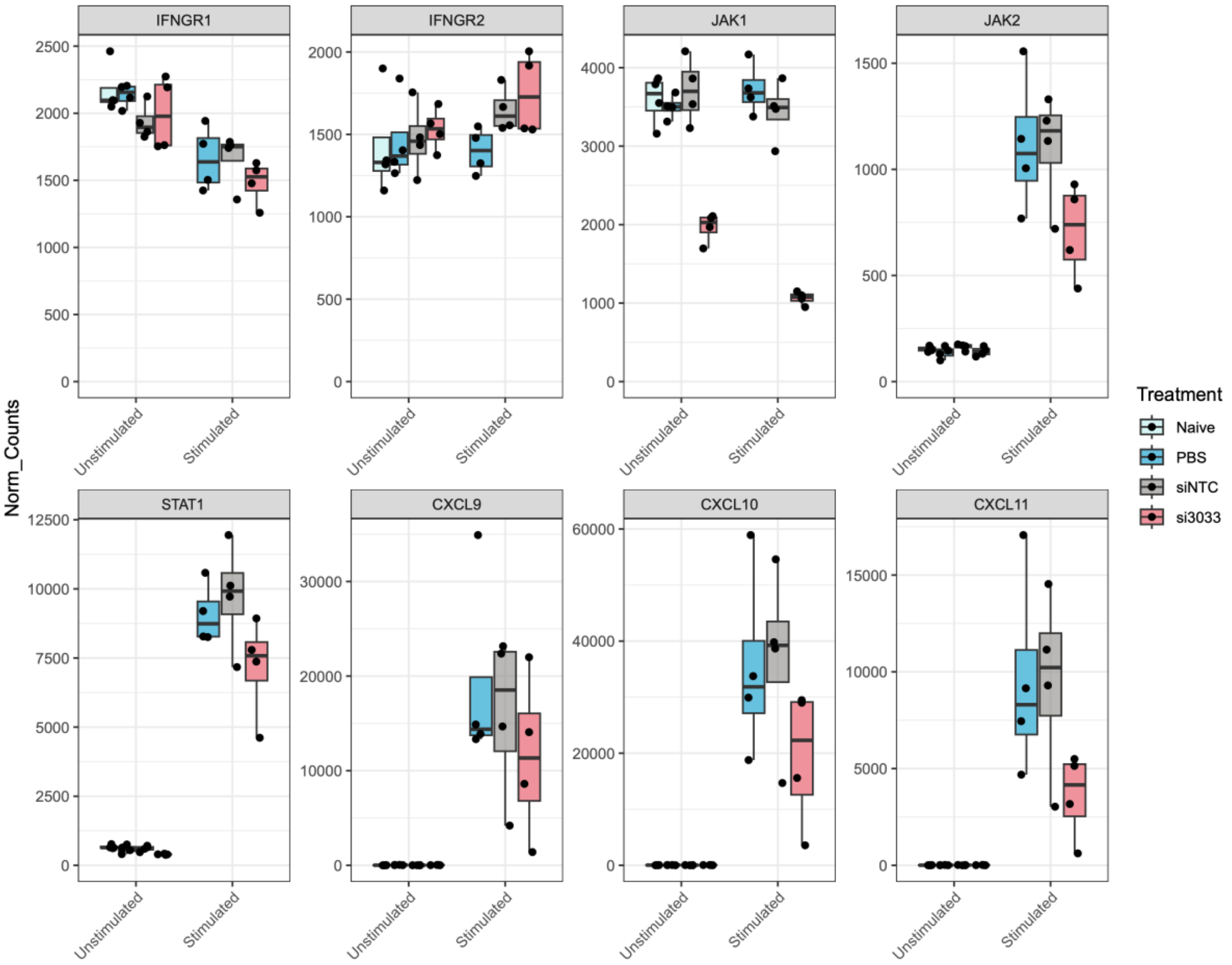

Supplementary Figure 9 | Boxplots of expression for genes involved in IFN-γ stimulation. IFN-γ strongly induces the expression of JAK2, STAT1, and the downstream chemokines CXCL9, CXCL10, and CXCL11, but has minimal impact on the expression of IFN-γ receptor subunits (i.e., IFNGR1 and IFNGR2) and JAK1. The y-axis shows the DESeq2 normalized counts.
